# Case Report: Successful management of hepatic injury secondary to mercury (II) oxide poisoning in a *Vulpes lagopus* with tail gland infection

**DOI:** 10.3389/fvets.2025.1724552

**Published:** 2026-01-21

**Authors:** BaoLian Yang, ZongSheng Qiu, ChengWei Wei, TianWen Ma

**Affiliations:** 1 Heilongjiang Key Laboratory for Laboratory Animals and Comparative Medicine, College of Veterinary Medicine, Northeast Agricultural University, Harbin, China; 2 Northeast Agricultural University Animal Clinical Teaching Hospital, Harbin, China

**Keywords:** mercury (II) oxide, Hydrargyri Oxydum Rubrum, liver injury, *Vulpes lagopus*, tail gland infection

## Abstract

A 6.08 kg female stray Arctic fox (*Vulpes lagopus*) of unknown age was presented with tail gland inflammation. Initial conventional therapy and subsequent tail amputation at a primary veterinary facility resulted in limited improvement. Subsequently, a topical medication red mercuric oxide (*Hydrargyri Oxydum Rubrum*) was applied for 4 weeks. Although the local infection showed signs of improvement, the fox subsequently developed progressive systemic signs, including anorexia, dark urine, and weight loss, prompting referral. Clinical examination revealed a large amount of cherry-red medication covering the wound. Hematological tests indicated elevated neutrophils and C-reactive protein (CRP), suggesting an inflammatory response. Serum biochemistry revealed elevated levels of alanine aminotransferase (ALT), aspartate aminotransferase (AST), alkaline phosphatase (ALP), and total bile acids (TBA), indicating hepatobiliary injury, alongside an elevated creatine kinase (CK) suggestive of abnormal muscle metabolism. The whole-blood mercury concentration was significantly elevated (4.7583 μg/L). Imaging findings included: ultrasound showing gallbladder sludge, abnormal liver parenchyma echogenicity, and indistinct kidney contours; X-ray revealed gastric gas, liver edge extending beyond the costal arch, blurred renal contours, and significantly increased density in the tail gland area. The Arctic fox was diagnosed with chronic topical mercury (II) oxide-induced mercury poisoning and secondary liver injury. The treatment regimen included: (1) removal of the topical medication and surgical debridement; (2) intravenous administration of reduced glutathione (hepatoprotection), ceftiofur sodium (anti-infective), and vitamin C (antioxidant); (3) oral administration of a mercury chelating agent (dimercaptosuccinic acid) and choleretics (ursodeoxycholic acid); and (4) intramuscular injection of appetite stimulants. After 4 weeks of systemic treatment, the fox’s abnormal biochemical parameters returned to normal, and the prognosis was good. This case addresses a specific gap in the diagnosis and treatment of heavy metal poisoning in wildlife. It provides a valuable reference for the clinical management of poisoning cases associated with topical mercury-containing wound medications.

## 1 Introduction

The traditional Chinese medicine red mercuric oxide (*Hydrargyri Oxydum Rubrum*), primarily composed of mercury (II) oxide, is soluble in dilute hydrochloric acid and is therefore strictly for external use only. Studies indicate that topical powders containing red mercuric oxide, at low concentrations, exert a mild stimulating effect on wounds, improving microcirculation and promoting wound repair, likely due to its effective bacteriostatic properties (1). Consequently, it has been used in wildlife medical management. However, prolonged or extensive application may lead to mercury absorption and subsequent intoxication, potentially causing hepatic and renal injury (2). However, the mercury element it contains can be absorbed into the bloodstream through the skin or wounds, irreversibly binding to sulfhydryl enzyme systems in the body. This inhibits cellular antioxidant capacity and induces oxidative stress and can lead to damage in parenchymal organs such as the liver and kidneys. Consequently, long-term or large-scale topical application increases the risk of mercury accumulation in tissues, causing systemic heavy metal poisoning (2, 3). Therefore, caution is advised when using red mercuric oxide in wildlife medicine.

The Arctic fox (*Vulpes lagopus*) is a mammalian species within the order *Carnivora*, family *Canidae*, and genus *Vulpes*. Arctic foxes possess well-developed tail glands. Tail gland inflammation is a prevalent condition in free-ranging Arctic foxes, typically manifesting with protrusion of the tail gland tissue—often accompanied by localized redness, swelling, or serous/purulent discharge (4, 5). If left untreated, it can progress to tissue necrosis, chronic pain, or secondary systemic infection due to the tail’s frequent contact with contaminated surfaces (6). Clinically, management strategies are stratified by disease severity: topical pharmacological agents are the first-line option for mild to moderate cases, while surgical excision is reserved for severe, recurrent inflammation or when glandular tissue has become irreversibly damaged. Notably, careful selection of topical drugs is critical, as prolonged or inappropriate use may lead to adverse effects like systemic toxicity (7).

This case report details the successful diagnosis and treatment of mercury (II) oxide poisoning and subsequent secondary hepatic damage in an Arctic fox (*Vulpes lagopus*). The poisoning was attributed to the repeated topical application of red mercuric oxide (*Hydrargyri Oxydum Rubrum*) for a persistent tail gland infection. To our knowledge, this is the complete documented case of iatrogenic poisoning caused by red mercuric oxide in an Arctic fox, primarily characterized by severe liver damage. The successful implementation of a comprehensive treatment regimen based on the oral chelating agent dimercaptosuccinic acid resulted in positive results. This study gives useful experience in the diagnosis and treatment of heavy metal poisoning in wildlife medicine, as well as an alert to doctors about the potential systemic toxic hazards of local use of mercury-containing medications.

## 2 Case description

### 2.1 Case presentation

**Figure 1:**
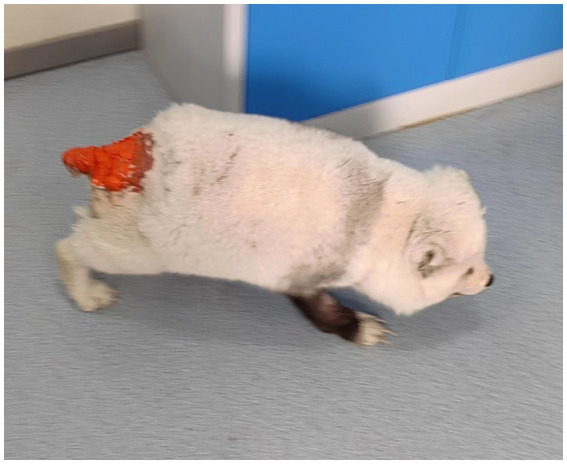
Image of Arctic fox with tail gland inflammation undergoing red mercuric oxide application.

A female Arctic fox weighing 6.08 kg was presented to the hospital. Due to its status as a stray animal, the exact age could not be determined. Pre-admission blood biochemistry and infectious disease screening revealed no significant abnormalities. In early March 2025, the fox developed a tail gland infection. Initial conventional topical therapy by the primary veterinarian yielded no significant improvement. For the four weeks preceding referral, the wound was treated with topical application of red mercuric oxide at a dosage of 10–15 mg/kg twice daily. Following each application, an Elizabethan collar was placed to prevent the fox from licking the affected area and ingesting the medication (Figure 1). However, the animal subsequently exhibited progressive signs including decreased appetite, dark urine, and emaciation, prompting referral to our institution. It was reported that the primary active ingredient of the topical agent was mercury (II) oxide. Physical examination revealed a body temperature of 38.4 °C, a heart rate of 105 beats per minute, and a body condition score of 6/9. The fox was lethargic, though ambulatory. Respirations were slightly rapid; no remarkable abnormalities were detected on pulmonary auscultation. Pupillary light reflexes were normal. Abdominal palpation revealed distension caudal to the costal arch. The skin exhibited reduced turgor, consistent with dehydration. The tail gland lesion was covered with a substantial amount of cherry red topical medication.

### 2.2 Investigations

**Figure 2:**
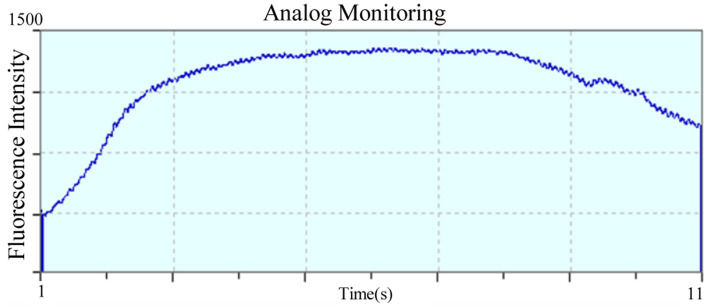
Results of mercury fluorescence intensity test in Arctic fox blood.

Blood routine examination was carried out on a fully automated veterinary five-part differential blood cell analyzer (BC-5000vet, Shenzhen Mindray Bio-Medical Electronics Co., Ltd., China). The results showed an increased neutrophil count and percentage (Table 1). C-reactive protein (CRP) levels were measured using a latex-enhanced immunoturbidimetric assay on an automated chemistry analyzer (Catalyst One^®^, IDEXX Laboratories, Shanghai, China). The results indicated that CRP was elevated (Table 2), indicating an inflammatory response likely associated with infection secondary to tail gland infection. Serum biochemical analysis was carried out utilizing a fully automated biochemical analyzer (Catalyst One^®^, IDEXX Maine Biological Products Trading (Shanghai) Co., Ltd., China). The results showed elevations in liver-related parameters including alanine aminotransferase (ALT), aspartate aminotransferase (AST), total bile acids (TBA), triglycerides (TG), alkaline phosphatase (ALP), cholesterol (CHOL), and blood urea nitrogen (BUN), suggestive of hepatobiliary injury. An increase in creatine kinase (CK) was also noted, which may be related to local muscle damage and catabolism associated with the tail gland infection (Table 3). Whole blood sample from the affected Arctic fox was analyzed for mercury using a thiourea mixture extraction method (Heilongjiang Huoyan Detection Technology Co., Ltd., China). The result indicated elevated blood mercury levels, measuring 4.7583 μg/L (Figure 2).

**Table 1:** Blood routine examination results of Arctic fox.

Parameter	Result	Unit	Reference scope	Prompt
White Blood Cell Count (WBC)	15.2	10^9^/L	6.00 ~ 17.00	
Neutrophil Count (NEU)	12.52	10^9^/L	3.62 ~ 12.30	↑
Lymphocyte Count (LYM)	1.73	10^9^/L	0.83 ~ 4.91	
Monocyte Count (MON)	0.59	10^9^/L	0.14 ~ 1.97	
Eosinophil Count (EOS)	0.25	10^9^/L	0.04 ~ 1.62	
Basophil Count (BASO)	0.11	10^9^/L	0.00 ~ 0.12	
Neutrophil Percentage (NEU)	82.4	%	52.0 ~ 81.0	↑
Lymphocyte Percentage (LYM)	11.4	%	12.0 ~ 33.0	
Monocyte Percentage (MONO)	3.9	%	2.0 ~ 13.0	
Eosinophil Percentage (EOS)	1.6	%	0.5 ~ 10.0	
Basophil Percentage (BASO)	0.7	%	0.0 ~ 1.3	
Red Blood Cell Count (RBC)	6.9	10^12^/L	5.10 ~ 8.50	
Hemoglobin (HGB)	133	g/L	110 ~ 190	
Hematocrit (HCT)	38.7	%	33.0 ~ 56.0	
Mean Corpuscular Volume (MCV)	56.2	fL	60.0 ~ 76.0	
Mean Corpuscular Hemoglobin (MCH)	19.2	pg	20.0 ~ 27.0	
Mean Corpuscular Hemoglobin Concentration (MCHC)	342	g/L	300 ~ 380	
Platelet Count (PLT)	247	10^9^/L	117 ~ 490	
Mean Platelet Volume (MPV)	7.3	fL	8.0 ~ 14.1	

↑ indicates a value higher than the reference value, ↓ indicates a value lower than the reference value.

**Table 2:** C-reactive protein (CRP) results of Arctic fox.

Parameter	Result	Unit	Reference scope	Prompt
C-Reactive Protein (CRP)	19.073	mg/L	<10	↑

↑ indicates a value higher than the reference value, ↓ indicates a value lower than the reference value.

**Table 3:** Results of blood biochemical examination of Arctic fox.

Parameter	Result	Unit	Reference scope	Prompt
Albumin (ALB)	33.7	U/L	23.00 ~ 40.00	
Total Protein (TP)	74.1	g/L	49.00 ~ 82.00	
Globulin (GLB)	40.4	g/L	19.00 ~ 45.00	
Albumin/Globulin Ratio (ALB/GLOB)	0.84		1.0–2.0	↓
Calcium (Ca)	2.72	mmol/L	1.98 ~ 3.00	
Creatine Kinase (CK)	203	U/L	10.00 ~ 200.00	↑
Blood Urea Nitrogen (BUN)	24.68	mmol/L	2.50 ~ 9.60	↑
Phosphorus (PHOS)	1.64	mmol/L	0.81 ~ 2.19	
Amylase (AMYL)	570	U/L	400 ~ 1,500	
Cholesterol (CHOL)	9.92	mmol/L	2.84 ~ 8.27	↑
Alanine Aminotransferase (ALT)	>650	U/L	5.00 ~ 125.00	↑
Total Bile Acid (TBA)	26.08	μmol/L	0.00 ~ 17.00	↑
Alkaline Phosphatase (ALKP)	221	U/L	17.00 ~ 212.00	↑
Creatinine (CREA)	90.4	μmol/L	28.00 ~ 159.00	
Triglyceride (TG)	2.24	mmol/L	0.00 ~ 1.13	↑
Aspartate Aminotransferase (AST)	91	U/L	0.00 ~ 50.00	↑

↑ indicates a value higher than the reference value, ↓ indicates a value lower than the reference value.

**Figure 3:**
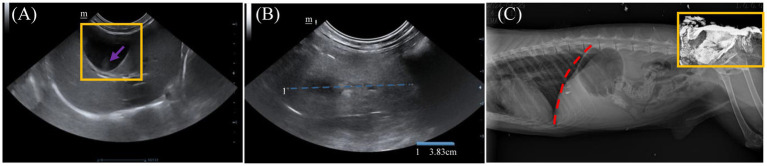
Imaging examination of Arctic fox. **(A)** Ultrasonographic image of the liver and gallbladder: The gallbladder is distended with a smooth wall and anechoic content (outlined in yellow). A small amount of irregularly shaped, medium-echoic biliary sludge is observed in the gravity-dependent region (purple arrowhead). **(B)** Ultrasonographic image of the kidney: the renal contours are indistinct, with a measured length of approximately 3.83 cm (blue dashed line). The renal cortex exhibits increased echogenicity, and the medullary architecture appears blurred. **(C)** Radiographic image: gas distension is visible within the stomach. The inferior liver margin extends beyond the costal arch (red dashed line). The renal structures are poorly defined, and the tail gland region shows markedly increased radiopacity (outlined in yellow).

Abdominal ultrasound examination was performed using a color Doppler ultrasound diagnostic system (GE LOGIQ F6™, General Electric Company, United States). The results showed a distended gallbladder with smooth walls and anechoic content; a small amount of medium echogenic biliary sludge with irregular morphology was observed in the gravity-dependent area. The common bile duct showed no significant dilation. The hepatic capsule was clear and smooth with sharp margins. The liver parenchyma exhibited a uniformly coarse, medium echogenic pattern, and the vascular structures appeared distinct without obvious abnormalities (Figure 3A). Renal ultrasonography demonstrated poor delineation of the renal contours; the long axis measured approximately 3.83 cm. The renal cortex showed increased echogenicity, and the medullary structure was blurred (Figure 3B). The remaining abdominal organs were unremarkable. Radiographic examination indicated no significant abnormalities within the thoracic cavity. Gastric gas distension was observed. The liver margin extended beyond the costal arch. The renal regions were poorly defined, and increased radiopacity was noted in the tail gland area, likely associated with trauma and topical medication administration (Figure 3C).

Based on the history, clinical signs, hematological and biochemical testing, diagnostic imaging, and blood mercury results, a comprehensive diagnosis of mercury (II) oxide poisoning due to prolonged topical application, with secondary hepatic injury, was made.

### 2.3 Treatment

The Arctic fox was hospitalized and underwent cleansing of the mercury (II) oxide -containing topical medication from the tail area. Necrotic tissue and foreign debris were then gently removed using curette and surgical scissors, followed by thorough wound irrigation and application of antiseptic, with the wound finally dressed under strict aseptic technique. Intravenous fluid therapy was administered, including reduced glutathione (300 mg/dose, Chongqing Yaoyou Pharmaceutical Co., Ltd., China), vitamin C (100 mg/dose, Jilin Huamu Animal Health Products Co., Ltd., China), and ceftiofur sodium (60 mg/dose, Hebei Yuanzheng Pharmaceutical Co., Ltd., China), to support liver function and provide anti-infective treatment. Mercury chelation was achieved with the administration of oral dimercaptosuccinic acid capsules (10 mg/kg, Shanghai Xinya Pharmaceutical Minxing Co., Ltd., China). Additionally, S-adenosylmethionine (50 mg/kg, dosed by body weight, Shandong Xinde Technology Co., Ltd., China) and ursodeoxycholic acid (10 mg/kg, dosed by body weight, Shandong Longchang Animal Health Products Co., Ltd., China) were administered orally for hepatoprotection and choleresis. Intramuscular injections included vitamin B complex (1 mL, Jilin Huamu Animal Health Products Co., Ltd., China) and compound butaphosphan injection (1 mL, Bayer AG, Germany) to stimulate appetite.

### 2.4 Outcome and follow-up

Following 5 days of systemic treatment, the Arctic fox’s clinical condition improved, with gradual return of appetite and water intake. After 7 consecutive days of intravenous fluid therapy, the fox’s status had largely returned to normal, and the tail gland infection showed marked improvement. Intravenous therapy was discontinued, and maintenance treatment continued with oral medications. After 4 weeks of sustained treatment, the previously abnormal biochemical parameters returned to normal levels (Table 4), and the prognosis was considered good.

**Table 4:** Re-examination results of blood biochemistry test for Arctic fox.

Parameter	Result	Unit	Reference scope	Prompt
Creatine Kinase (CK)	54	U/L	10.00 ~ 200.00	
Blood Urea Nitrogen (BUN)	4.74	mmol/L	2.50 ~ 9.60	
Cholesterol (CHOL)	4.39	mmol/L	2.84 ~ 8.27	
Alanine Aminotransferase (ALT)	82	U/L	5.00 ~ 125.00	
Total Bile Acid (TBA)	6.92	μmol/L	0.00 ~ 17.00	
Alkaline Phosphatase (ALKP)	65	U/L	17.00 ~ 212.00	
Triglyceride (TG)	0.58	mmol/L	0.00 ~ 1.13	
Aspartate Aminotransferase (AST)	27	U/L	0.00 ~ 50.00	

## 3 Discussion

Mercury is a heavy metal with significant biotoxicity, and its toxicity is closely related to its chemical form. The mercury (II) oxide discussed in this case is an inorganic compound containing divalent mercury (Hg^2+^) (8). Mercury ions can enter the body through skin or mucous membrane contact and bind irreversibly to highly reactive groups like sulfhydryl (-SH) groups in different proteins and enzymes within cells. This binding inhibits the activity of key enzymes such as cytochrome oxidase and succinate dehydrogenase (9, 10). This widespread inhibition of the enzyme system constitutes the molecular mechanism by which mercury (II) oxide induces multi-organ toxicity, which can clinically manifest as acute renal failure, liver injury, and central nervous system dysfunction (11).

In this case, the Arctic fox developed a characteristic cherry-red, hard medicinal crust due to the long-term topical application of red mercuric oxide for a tail gland infection. This crust served as a continuous source of mercury exposure. Although mercury poisoning is uncommon in wild animals and can be easily overlooked, the distinctive cherry-red color of the topical agent (mercury (II) oxide) can provide a crucial diagnostic clue, helping to identify the etiology. The majority of cases of mercury (II) oxide poisoning had a clear history of mercury-containing medication use or exposure to a relevant living environment. When animals develop unexplained symptoms in the kidneys, liver, or neurological system, mercury poisoning should be suspected. Notably, distinct renal compensatory systems are found in dogs, foxes, and cats. In particular, biochemical indicators such as creatinine typically become notably elevated only when 75% or more of renal function is lost, which meets the diagnostic criteria for renal failure (12). According to reports, the main symptoms of early-stage chronic methylmercury poisoning in adult cats are neurological, with signs of organ damage showing up later (13). During its initial stay at the rescue center, the Arctic fox’s blood biochemistry indications fell within normal levels. Biochemical markers showed signs of liver damage when mercury (II) oxide was used topically to alleviate tail gland inflammation. However, there was no increase in blood creatinine (CREA) or other pertinent indicators, and renal damage remained below the functional compensation threshold, only showing up on ultrasound imaging as increased cortical echogenicity and muddled medullary structure. The discrepancy between imaging findings and biochemical parameters essentially reflects the difference between structural kidney damage and functional compensation. The kidneys possess substantial functional reserve; even when partial nephron impairment leads to abnormal imaging manifestations, the remaining healthy nephrons can still maintain overall filtration function through compensatory mechanisms, thereby keeping biochemical indicators such as blood urea nitrogen and creatinine within normal ranges. Thus, imaging alterations can be regarded as early warning signs of renal injury, indicating the presence of parenchymal pathology while renal function remains in a compensated state. Therefore, for species with strong renal compensatory capacity, a comprehensive assessment integrating imaging, pathology, and toxicological testing is essential to avoid misdiagnosis due to reliance on a single indicator. Additionally, the hematological information in this case is derived from blood routine examination and serum biochemical analysis; no blood smears were used.

This instance exhibits unique exposure and species specificity traits. In terms of tissue accumulation patterns, research verifies that the distribution of mercury in foxes follows the order: liver>kidney>muscle>brain (14), whereas long-term use of mercury-containing cinnabar in rats causes primary accumulation in kidneys and brains, resulting in pathological alterations (15). In terms of exposure routes, bioaccumulation in the food chain is the main cause of mercury poisoning in wildlife. For example, methylmercury poisoning can cause neurological symptoms like ataxia in wild mink that consume contaminated prey (16). On the other hand, continuous topical application of mercury (II) oxide preparations resulted in skin absorption poisoning in this case, which illustrates iatrogenic exposure in an artificial rescue scenario. This finding is consistent with a reported case of iatrogenic mercury exposure causing nephrotic syndrome in a domestic cat (13). Together, these cases underscore the need for increased vigilance regarding the risk of systemic toxicity from topical mercury-containing drugs in veterinary clinical practice.

The diagnosis of mercury poisoning is primarily confirmed by detecting mercury levels in whole blood, urine, or hair; fluorescent probes represent an alternative diagnostic method (17–19). Regarding treatment, the clinical management of mercury poisoning involves specific chelation therapy combined with symptomatic support. The specific antidotal strategy relies on the use of sulfhydryl chelating agents. These agents utilize their active sulfhydryl groups to bind with mercury ions in the body, forming stable, water-soluble complexes. This process displaces mercury from enzyme systems, allowing for its excretion in urine and restoring enzymatic function (20). In this case, dimercaptosuccinic acid was selected as the oral heavy metal antidote. Both intravenous infusion and oral administration of this medication have been shown to significantly lower blood mercury levels and alleviate clinical symptoms in dogs suffering from mercury poisoning (21, 22). The oral route is more appropriate for wild animals that are challenging to confine because of their ease of use and low risk of side effects. In contrast, other chelating agents, such as dimercaprol, require intramuscular injection and carry risks of local irritation, while sodium dimercaptopropanesulfonate requires intravenous administration, which involves more complex operational requirements and thus has limited application in wildlife rescue scenarios (23). Furthermore, the sensitivity of various types of mercury to chelating agents differs, with dimercaprol showing better chelation efficiency for inorganic mercury. The treatment plan was suitable in this situation since mercury (II) oxide induced inorganic mercury toxicity. It is intended that this will serve as a useful guide for the identification and management of inorganic mercury toxicity in wildlife.

## 4 Conclusion

This case report describes the diagnosis and treatment of iatrogenic mercury poisoning and resulting liver impairment in an Arctic fox induced by long-term topical mercury (II) oxide usage. By incorporating prior studies on animal mercury poisoning, the study highlights the possible dangers associated with topical mercury-containing treatments, species-specific intoxication characteristics, and the efficacy of an integrated treatment strategy centered on oral dimercaptosuccinic acid. For future wildlife rescue and therapeutic practice, it is critical to improve heavy metal risk evaluations in topical treatments, develop a multidimensional diagnostic framework, and optimize treatment techniques based on species differences and mercury speciation. This will provide more thorough theoretical and practical assistance for the prevention, control, and clinical treatment of heavy metal poisoning in animals.

## Data Availability

The original contributions presented in the study are included in the article/supplementary material, further inquiries can be directed to the corresponding authors.
